# Supplementary material for: Hydrogen Sulfide-Evoked Intracellular Ca2+ Signals in Primary Cultures of Metastatic Colorectal Cancer Cells
Source: Cancers (Basel). 2020 Nov 11;12(11):3338. doi: 10.3390/cancers12113338 (PMC7696676; doi:10.3390/cancers12113338)

Supplementary Materials: Hydrogen Sulphide-Evoked Intracellular Ca^{2+} Signals in Primary Cultures of Metastatic Colorectal Cancer Cells

Pawan Faris, Federica Ferulli, Mauro Vismara, Matteo Tanzi, Sharon Negri, Agnese Rumolo, Kostantinos Lefkimmiatis, Marcello Maestri, Mudhir Shekha, Paolo Pedrazzoli, Gianni Francesco Guidetti, Daniela Montagna and Francesco Moccia

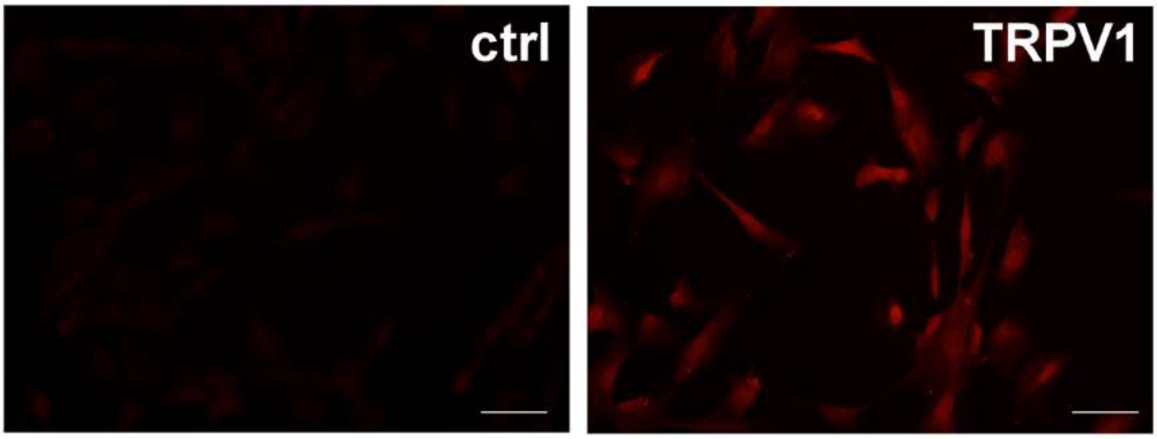

Figure S1. Representative immunofluorescence analysis of TRPV1 expression in mCRC cells. Cells were seeded onto glass coverslips and probed to detect the expression of TRPV1 (red). Analysis at 200x magnification. The negative control sample (ctrl) was obtained by staining with TRITC-labeled secondary antibody in the absence of the primary antibody. Scale bar: 100 μm.

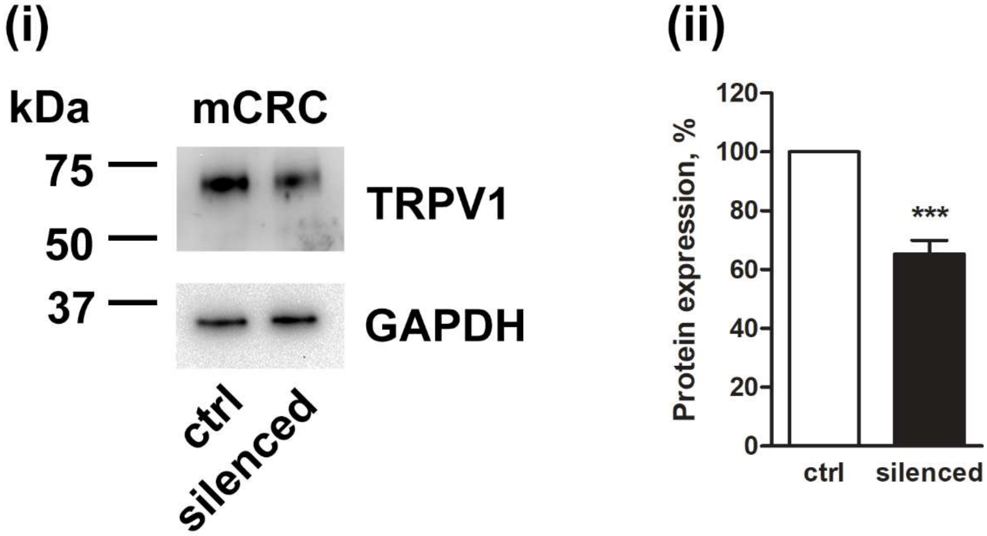

Figure S2. Genetic silencing of TRPV1 in silenced mCRC cells. Immunoblotting analysis of TRPV1 expression in mCRC silenced by siRNA. Representative immunoblots are reported in (**i**), where GAPDH staining was for equal loading control. Quantification of the results performed by densitometric scanning is reported in (**ii**), as percentage of protein expression compared to control (ctrl). Results are the mean ± SD of three different experiments. Student's t-test has been used for statistical comparison. *** $p < 0.005$.

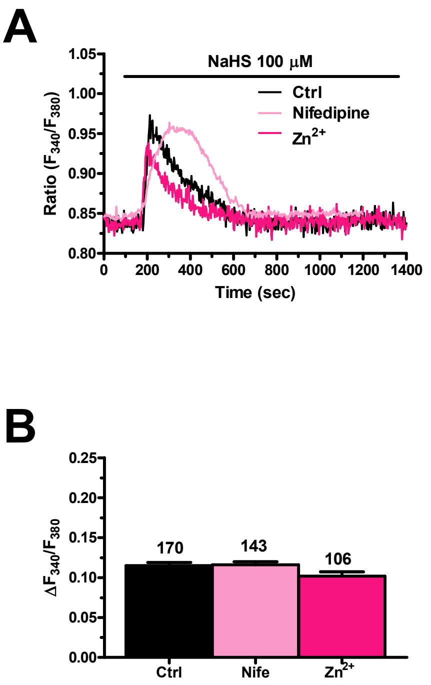

Figure S3. The pharmacological blockade of voltage-gated Ca^{2+} channels does not affect NaHS-evokes intracellular Ca^{2+} signals in CRC and non-neoplastic cells. **A**, NaHS (100 μM) evoked intracellular Ca^{2+} signals in mCRC cells in the absence (Ctrl) and in the presence of nifedipine (10 μM, 30 min) or Zn^{2+} (100 μM, 15 min). **B**, mean ± SE of the amplitude of the peak Ca^{2+} response induced by NaHS under the designated treatments.

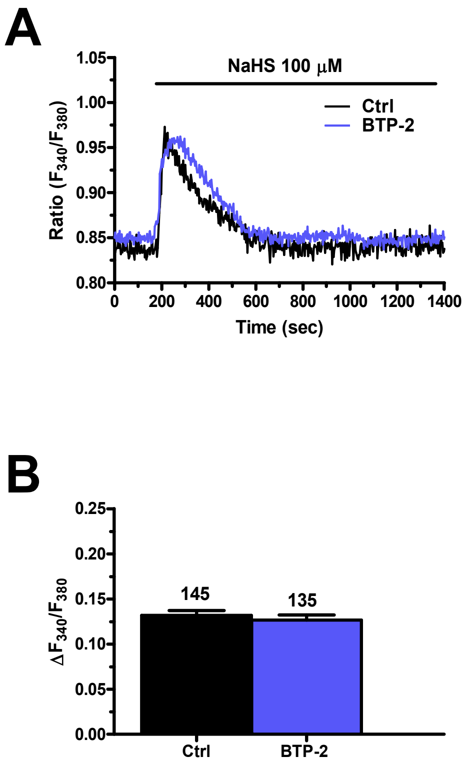

Figure S4. The pharmacological blockade of SOCE does not affect NaHS-evokes intracellular Ca^{2+} signals in CRC and non-neoplastic cells. **A**, NaHS (100 μM) evoked intracellular Ca^{2+} signals in mCRC cells in the absence (Ctrl) and in the presence of BTP-2 (20 μM, 30 min), a selective SOCE inhibitor. **B**,

mean ± SE of the amplitude of the peak Ca^{2+} response induced by NaHS under the designated treatments.

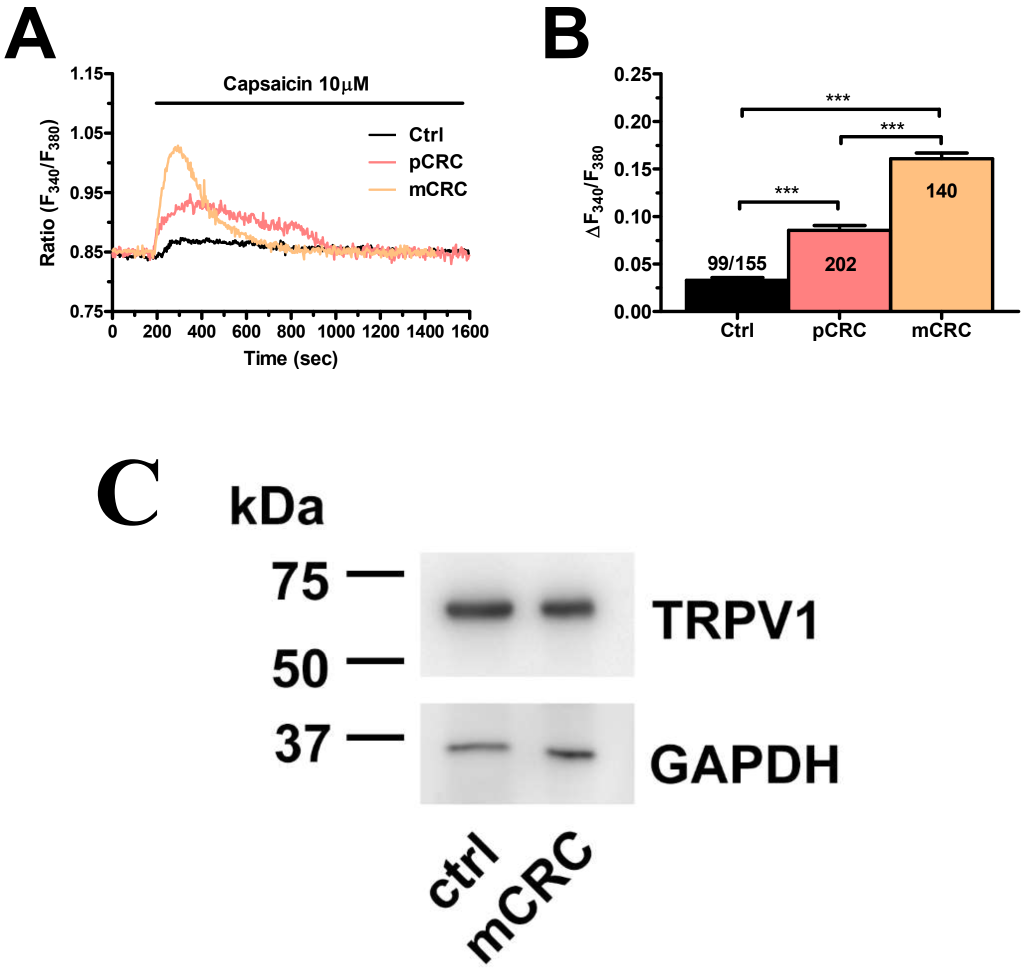

Figure S5. TRPV1-mediated extracellular Ca^{2+} entry is larger in mCRC cells. **A**, capsaicin-evoked intracellular Ca^{2+} signals which in non-neoplastic (Ctrl), pCRC and mCRC cells. Capsaicin was administered at 10 μM. **B**, mean ± SE of the amplitude of the peak Ca^{2+} response induced by capsaicin in the three different cell types. One-way Anova analysis followed by the post-hoc Dunnett test was used for Statistical comparison. *** $p \leq 0.001$. **C**, immunoblotting analysis of TRPV1 expression in non-neoplastic (ctrl) and mCRC cells. Total lysates (5 μg) were separated by SDS-PAGE, blotted on PVDF membrane and stained with anti TRPV1 antibody. Subsequent reprobing with anti-GAPDH antibody was performed as an equal loading control.

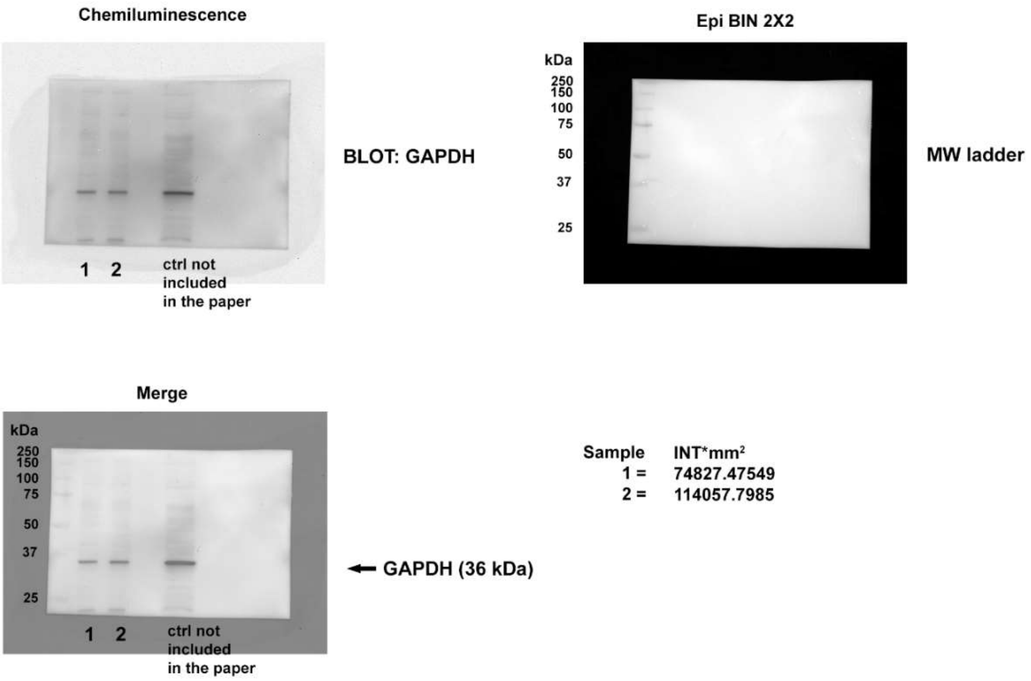

Figure S6. Uncropped blots corresponding to Western blot in the Figure 8A in the main text.

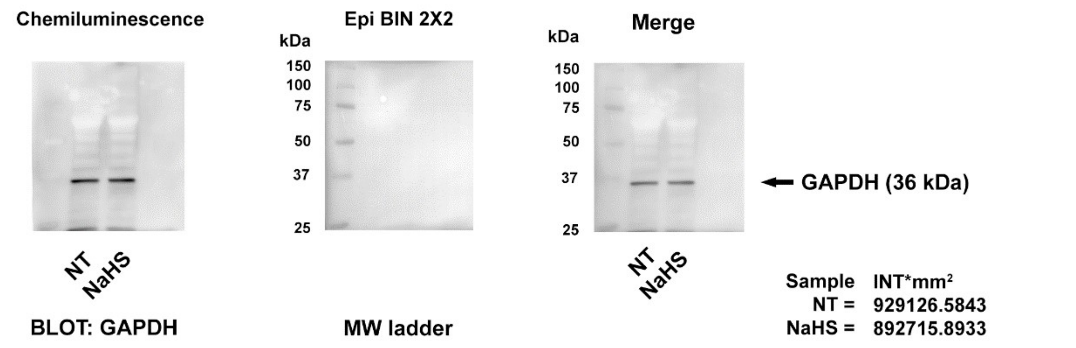
Chemiluminescence
Epi BIN 2X2
Merge
kDa
150
100
75
50
37
25
GAPDH (36 kDa)
NT
NaHS
BLOT: GAPDH
MW ladder
Sample INT*mm²
NT = 929126.5843
NaHS = 892715.8933

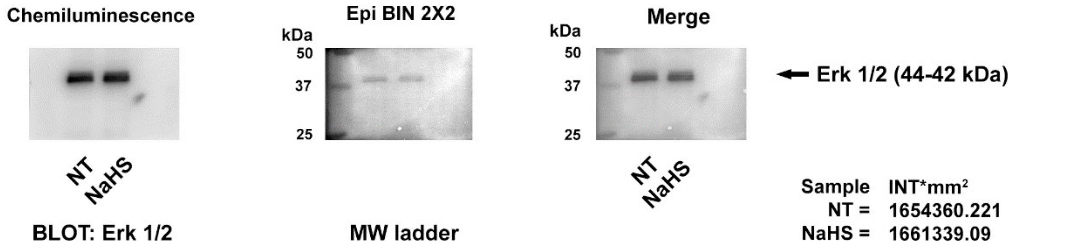
Chemiluminescence
Epi BIN 2X2
Merge
kDa
50
37
25
Erk 1/2 (44-42 kDa)
NT
NaHS
BLOT: Erk 1/2
MW ladder
Sample INT*mm²
NT = 1654360.221
NaHS = 1661339.09

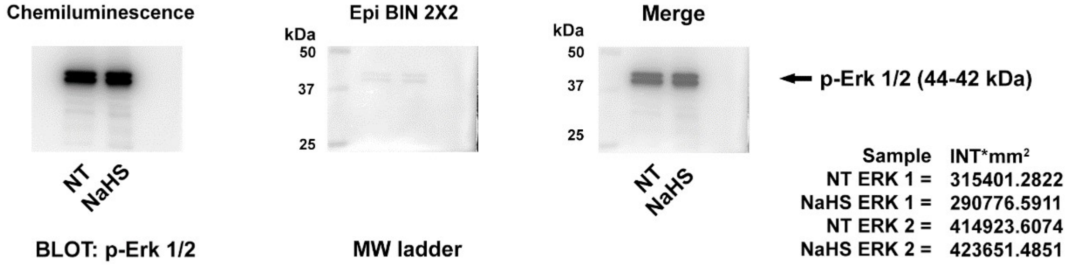
Chemiluminescence
Epi BIN 2X2
Merge
kDa
50
37
25
p-Erk 1/2 (44-42 kDa)
NT
NaHS
BLOT: p-Erk 1/2
MW ladder
Sample INT*mm²
NT ERK 1 = 315401.2822
NaHS ERK 1 = 290776.5911
NT ERK 2 = 414923.6074
NaHS ERK 2 = 423651.4851

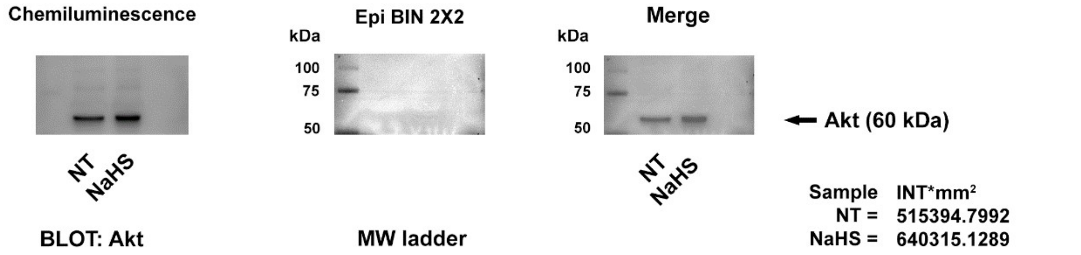
Chemiluminescence
Epi BIN 2X2
Merge
kDa
100
75
50
Akt (60 kDa)
NT
NaHS
BLOT: Akt
MW ladder
Sample INT*mm²
NT = 515394.7992
NaHS = 640315.1289

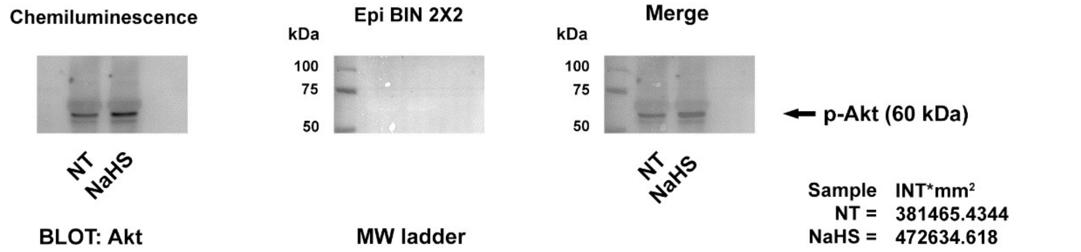
Chemiluminescence
Epi BIN 2X2
Merge
kDa
100
75
50
p-Akt (60 kDa)
NT
NaHS
BLOT: Akt
MW ladder
Sample INT*mm²
NT = 381465.4344
NaHS = 472634.618

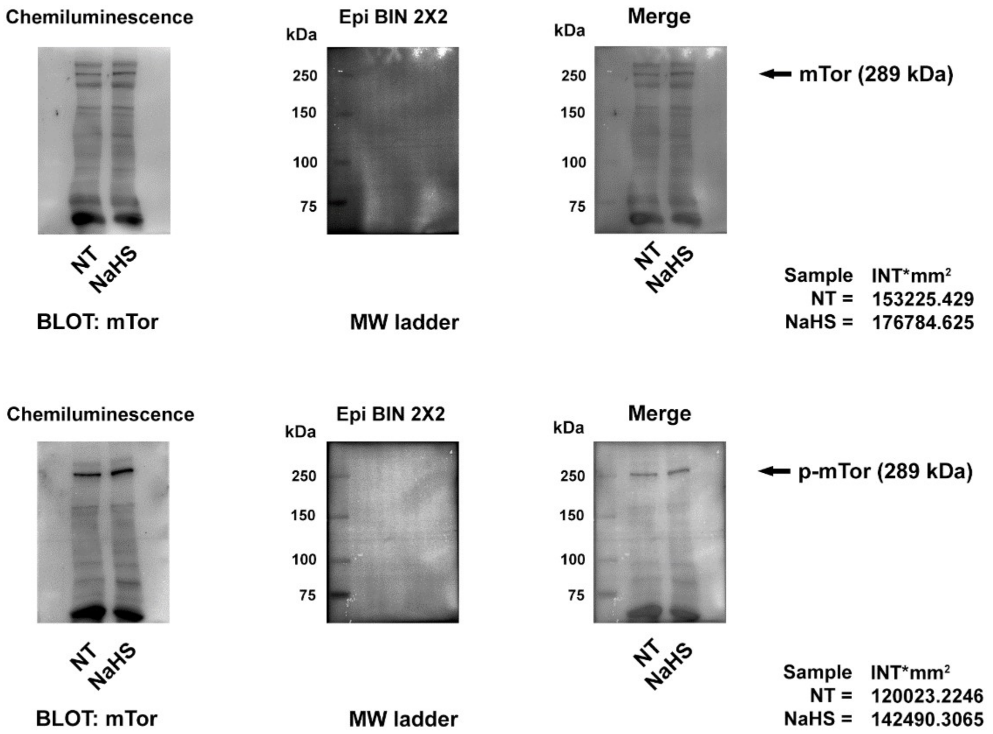

Figure S7. Uncropped blots corresponding to Western blot in the Figure 14 in the main text.

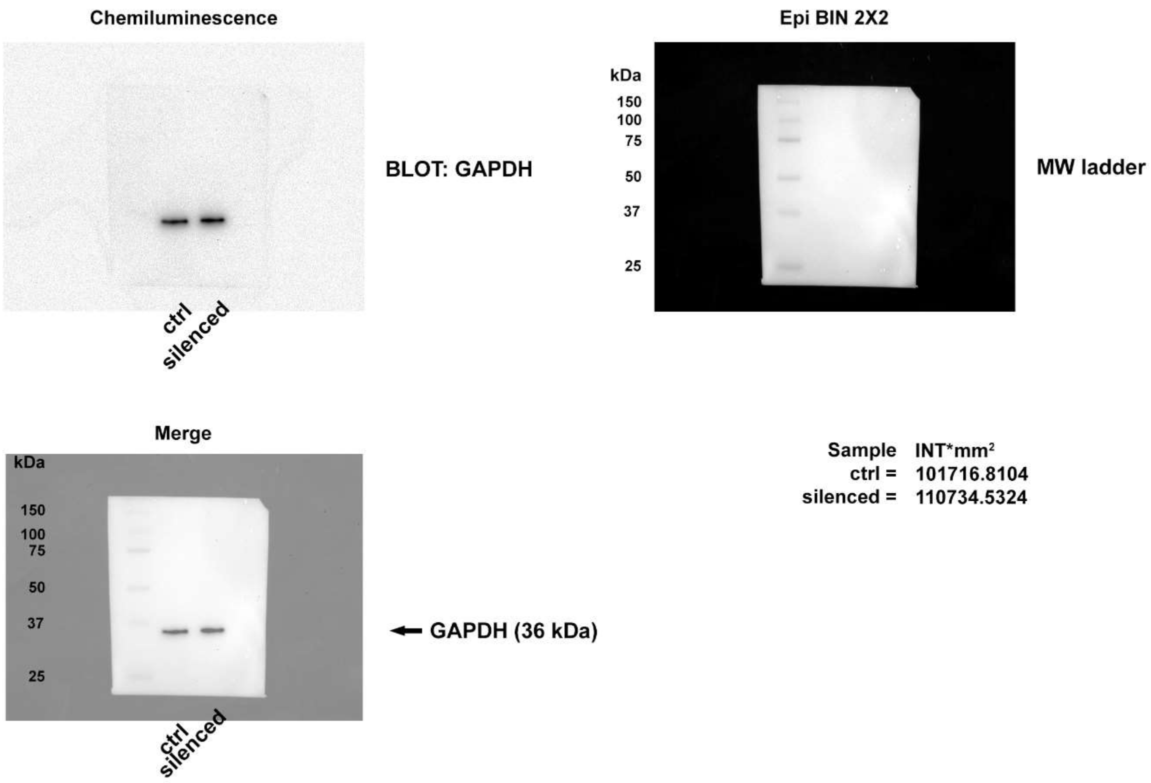

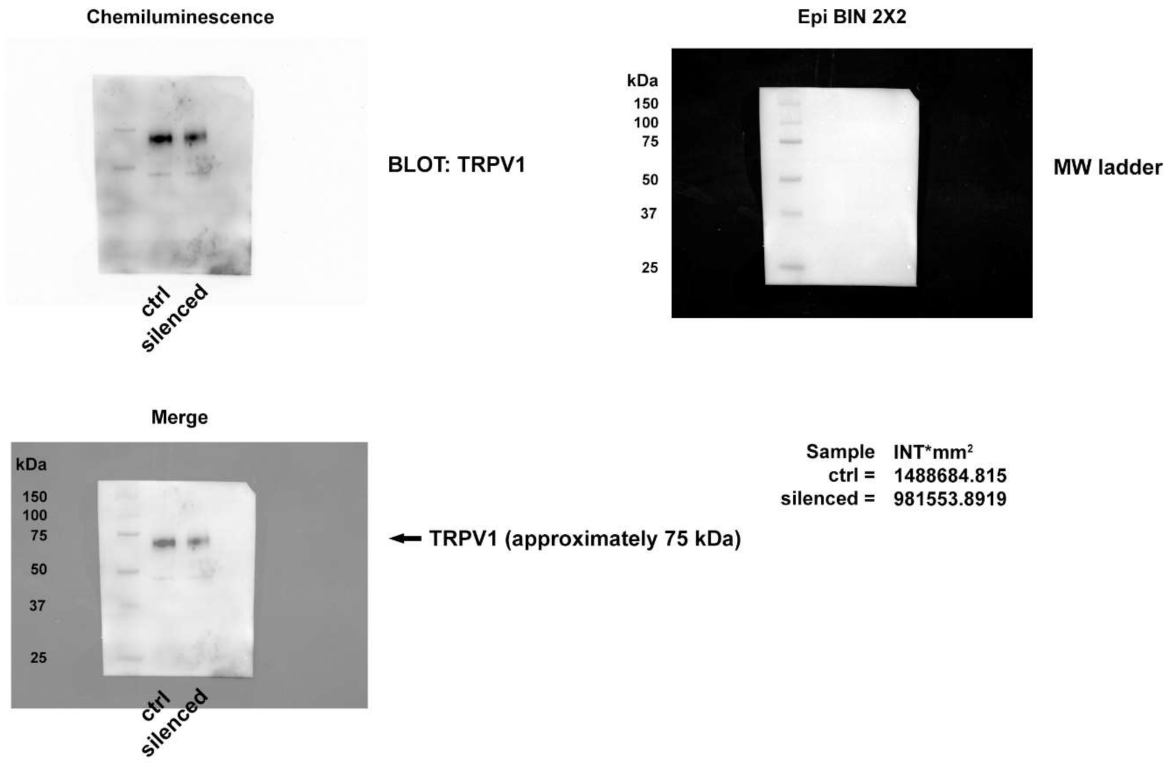

Figure S8. Uncropped blots corresponding to Western blot in the Figure S2 shown above.

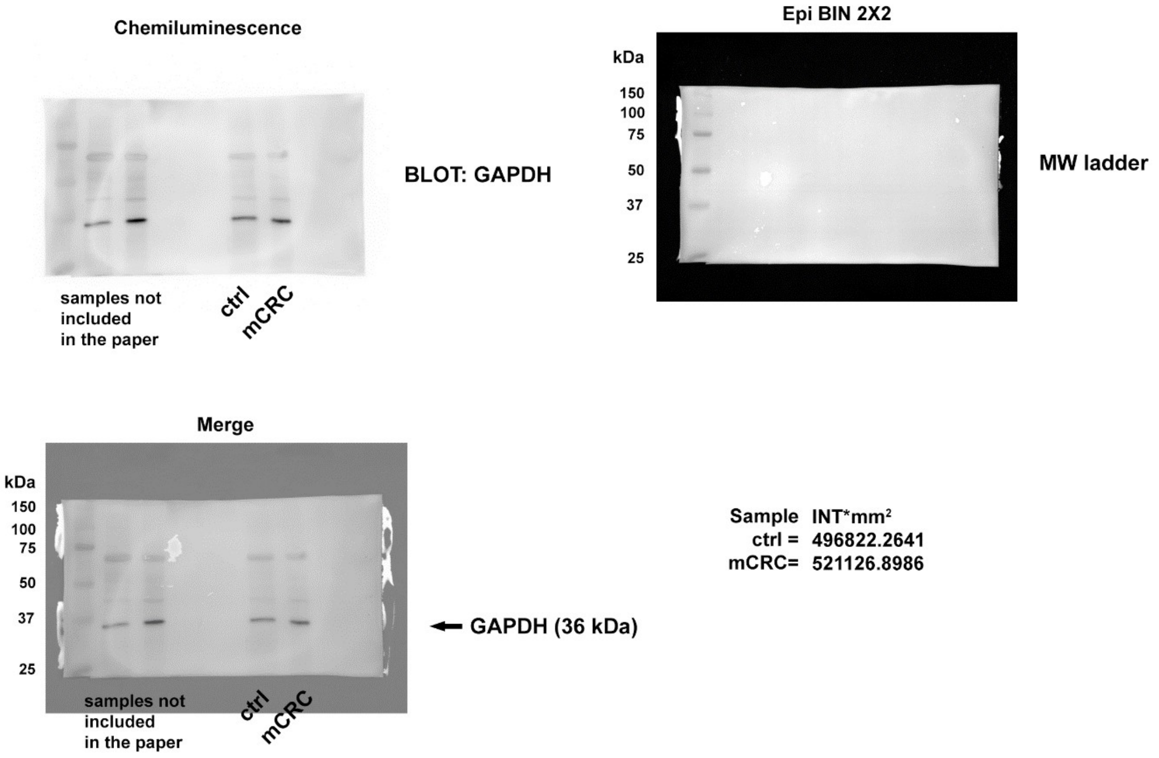

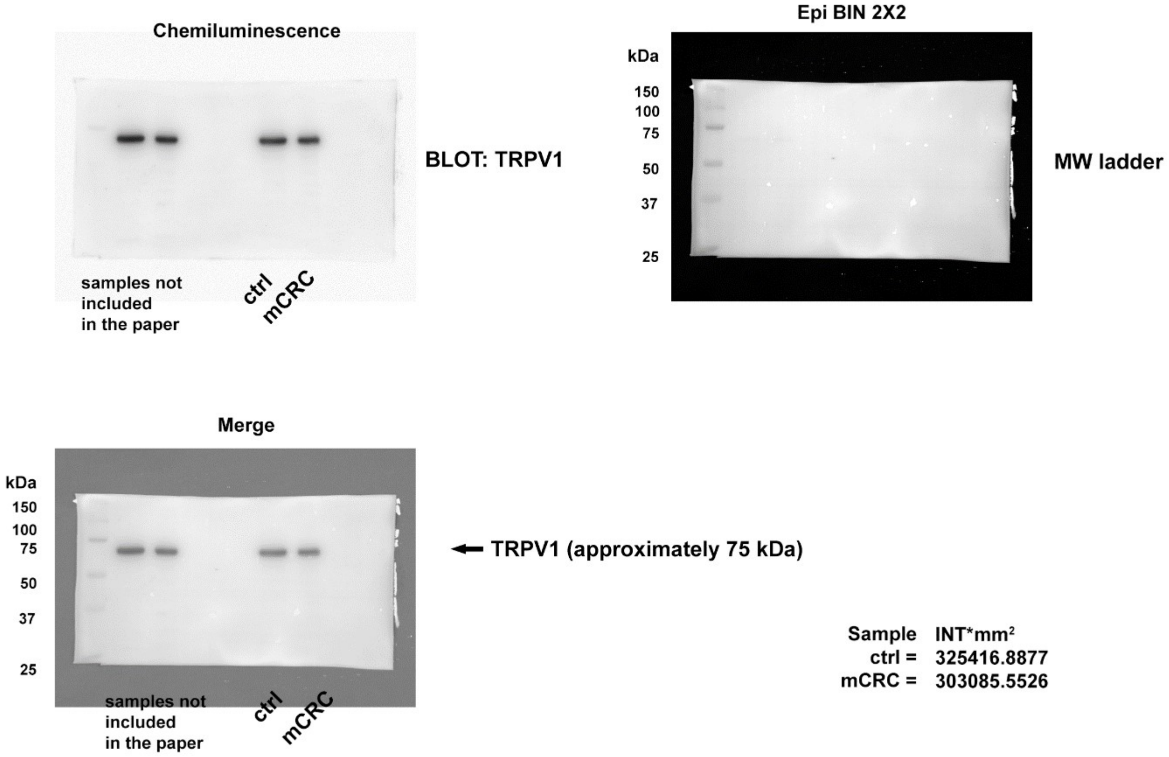

Figure S9. Uncropped blots corresponding to Western blot in the Figure S5 shown above.

© 2020 by the authors. Licensee MDPI, Basel, Switzerland. This article is an open access article distributed under the terms and conditions of the Creative Commons Attribution (CC BY) license (http://creativecommons.org/licenses/by/4.0/).

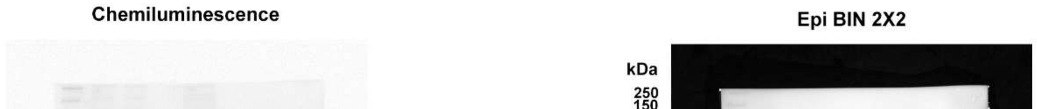

Supplement: Supplementary file 1 [file cancers-12-03338-s001.pdf]
